# Stability of Mental Toughness, Sleep Disturbances, and Physical Activity in Patients With Multiple Sclerosis (MS)—A Longitudinal and Pilot Study

**DOI:** 10.3389/fpsyt.2018.00182

**Published:** 2018-05-17

**Authors:** Dena Sadeghi Bahmani, Leila Esmaeili, Vahid Shaygannejad, Markus Gerber, Juerg Kesselring, Undine E. Lang, Edith Holsboer-Trachsler, Serge Brand

**Affiliations:** ^1^Center for Affective-, Stress- and Sleep Disorders, Psychiatric Clinics (UPK), University of Basel, Basel, Switzerland; ^2^Sleep Research Center, Kermanshah University of Medical Sciences, Kermanshah, Iran; ^3^Department of Psychology, Education & Psychology Faculty, University of Isfahan, Isfahan, Iran; ^4^Isfahan Neurosciences Research Center, Alzahra Research Institute, Isfahan University of Medical Sciences, Isfahan, Iran; ^5^Sport Science Section, Department of Sport, Exercise and Health, University of Basel, Basel, Switzerland; ^6^Kliniken Valens, Valens, Switzerland; ^7^Kermanshah University of Medical Sciences (KUMS), Sleep Disorders Research Center and Substance Abuse Prevention Research Center, Kermanshah, Iran

**Keywords:** multiple sclerosis, mental toughness, sleep, physical activity, long-term

## Abstract

**Background:** Previous research of patients with multiple sclerosis (MS) focused prevalently on fatigue, depression, and cognitive dysfunction during the clinical course. By contrast, research on the longer-term characteristics of physical activity (PA), psychological functioning, and sleep problems is scarce. The aims of the present study were therefore to examine changes in PA, mental toughness (MT) as a proxy of psychological functioning, and sleep disturbances over a 2-year period of time after disease onset.

**Methods:** A total of 18 patients with diagnosed MS (mean age: *M* = 34.29 years) took part in this longitudinal study. First, 1–4 weeks after the first symptoms, a neurologist diagnosed the MS. Second, they completed a series of questionnaires covering socio-demographic data, PA, MT, and sleep disturbances. Third, the same questionnaires were completed again 2 years later (follow-up). Last, a neurologist assessed the degree of disability with the Expanded Disability Status Scale (EDSS).

**Results:** Two years after MS onset, patients had lower levels of vigorous PA, but no statistically significant changes in moderate PA were observed. Further, walking time increased and sedentary time decreased. Patients with sleep disturbances at disease onset also reported poor sleep 2 years later. MT scores remained stable over time. EDSS scores worsened, though, change in EDSS was not associated with PA, MT, or sleep.

**Conclusions:** Two years after disease onset, patients with MS reported similar MT levels and sleep disturbances. PA shifted from vigorous PA toward walking and a less sedentary lifestyle, while moderate PA remained unchanged. The pattern of results of the present pilot study suggests that at the early stage of the MS course, there is no obstacle for being physically active, nor did sleep and MT as a proxy of psychological functioning decrease in a substantial way.

## Introduction

Multiple sclerosis (MS) is an autoimmune, chronic, inflammatory, and demyelinating neurodegenerative disease (1–3), and as with all neurodegenerative diseases such as Alzheimer's disease, Parkinson's disease, or Huntington's disease, physical and psychological functioning are at high risk to be impaired. As regards MS, several studies reported that patients with diagnosed MS were at increased risk to suffering from sleep and cognitive issues (4). With regard to physical functioning, impaired gross and fine motor behavior, and reduced physical activity (PA) levels are observed (5); with regard to psychological functioning, increased levels of anxiety and depression have been reported (6–9). Further, patients with MS often report sleep disturbances (9–12), fatigue (13–15), and paraesthesia (1, 3), all of which may contribute to decreased quality of life.

Patients, their relatives, and their health care providers are interested in knowing more about the outlook of PA, psychological functioning and sleep as easily observable and detectable signals of quality of life. Accordingly, in the present study, we focused on the question how PA, mental toughness as a proxy of psychological functioning and sleep developed over time from disease onset until 2 years later.

As regards PA, there is extant literature on the cross-sectional association between the degree of disease disability and PA impairment (16–19), and a generally lower PA intensity and frequency compared to healthy people has been described (20). Specifically, Veldhuijzen van Zanten et al. (20) showed that patients with MS spend more time in sedentary activities compared to healthy people. In this view, there is increasing evidence that PA interventions have the potential to improve fatigue, depression (5, 21), anxiety (21), paraesthesia (5), and fitness (21, 22); this holds particularly true, if the minimum of regular PA per week ([30 min of moderate PA intensity for at least 2 times per week (23, 24) is taken into account. However, while the amount of intervention studies do increase, which show that regular PA is a safe method and has the power to increase physical and psychological well-being (19, 25, 26) also among those patients with severe mobility disability (27), non-interventional longitudinal research is scarce: For instance, Motl et al. (18) showed that PA behavior before disease onset predicted PA behavior after disease onset, suggesting thus that PA behavior follows a rather robust pattern. Further, Motl and McAuley (28) showed that a decrease in PA after disease onset was associated with an increase in physical impairment. In the present study, we expanded upon previous research in the sense that we distinguished between activities with different intensities (moderate and vigorous PA; walking and sedentary behavior) and that we explored, if and to which extent these categories changed 2 years after the disease onset.

As regards psychological functioning among patients with MS, research has extensively focused on symptoms of depression (6–8), anxiety (6, 29), alexithymia (30), or psychiatric disorders in general (31). Again, while cross-sectional studies are frequent, this is less the case for longitudinal data: Berzins et al. (32) showed that a 2-week incidence of depression was 0.019 for female, and 0.044 for male patients, thus, incidence rate seemed quite high. Contributing risk factors were higher fatigue, lower mobility, lower self-esteem, and lower self-efficacy, along with less favorable coping styles. Thus, incidence of depression was related to both illness-related and psychological factors. More specifically, in the previous study and the current baseline study (33), we showed that mental toughness (MT) scores of patients with diagnosed MS at disease onset were as high as MT scores of young adolescents. These results therefore suggested that a psychological decline as a pre-clinical sign of disease onset seemed very unlikely. Further, in our opinion, the concept of MT is particularly suitable to assess psychological functioning, as MT covers a broad variety of dimensions such as self-esteem, self-efficacy, coping styles, confidence, and social behavior. Mental toughness is a psychological construct to describe the capacity of person to be continuously successful in facing life difficulties. MT consists of the following dimensions: Control (emotions and own life), Commitment (to one's achievements and aims), Challenge (changes in life not taken as challenges and not as threats), and Confidence (in other people and in one's own abilities; (34, 35). Thus, MT refers to the tendency to understand threats and stress as opportunities to thrive (36), actively to seek and cope with challenges (37), and successfully to deal with setbacks and difficulties (34, 38). Accordingly, MT encompass a range of cognitive-emotional processes involved in coping with stress, motivation, self-efficacy, unpredictable situations, and social circumstances. Lin et al. (39) reported in their recent review that MT is an umbrella term that entails positive psychological resources which are associated with a broad range of mental health outcomes. Specifically, Lin et al. (39) mentioned the “mental toughness advantage,” as MT traits are associated with positive goal traits, more efficient coping strategies and positive outcomes in education and mental wellbeing. In this view, studies of non-clinical samples showed that higher MT scores were associated with lower scores of symptoms of depression and stress (40–44), and accordingly, the second aim of the present study was to explore, if and to what extent MT scores changed in MS patients over time from disease onset until 2 years later.

With regard to sleep, Strober (14) reported that sleep problems in MS have gone fairly unrecognized until recently, and that poor sleep was associated with increased fatigue and depression (9). Caminero and Bartolomé (10) reported that sleep disturbances seemed to be more frequent among patients with MS, and that the underlying mechanisms might be related to Periodic Limb Movements (PLM), restless legs syndrome (RLS), respiratory issues, or circadian rhythm disorders. Next, Antonijevic and Steiger (45) showed that compared to healthy controls and before treatment with corticosteroids, female patients with MS had more slow-wave sleep and less Stage-2-sleep, as assessed via polysomnography. Further, Braley and Boudreau (46) summarized in their review, that research on sleep in patients with MS was scarce, that longitudinal data were missing and that the scientific community mostly focused on daytime fatigue and further chronic symptoms in MS. By contrast, Sadeghi Bahmani et al. (33) recently showed that in patients with diagnosed MS at disease onset, sleep quality was equal to the sleep quality of adolescents and young adults, suggesting therefore that at disease onset sleep quality was not more or less impaired, compared to people without MS. However, longitudinal data on change or stability of sleep are missing. Accordingly, the third aim of the present study was to compare sleep quality at disease onset and 2 years later.

Within the scope of the present article, four hypotheses and one exploratory research question were formulated. First, following Motl et al. (18), we hypothesized that PA at disease onset was positively associated with PA 2 years later. Second, following Motl and McAuley (28), we assumed that PA would decrease as a function of increased disability. Third, following Veldhuijzen van Zanten et al. (20), we expected that sedentary behavior would increase over time. Fourth, following Berzins et al. (32), we assumed that MT scores would decrease over time. The exploratory research question addressed the issue, if and to what extent sleep disturbances remained stable over a 2-year period from disease onset until follow-up. Thus, the present study will shed more light on the course of PA, psychological functioning and sleep, dimensions, which are regarded as reliable signs of the course of illness.

## Methods

### Procedures

Patients with MS at disease onset (33) were contacted 2 years later to participate in the present follow-up study. The aims of the follow-up study and the confidential data management were fully explained; thereafter, patients gave their written informed consent. Participants completed questionnaires covering socio-demographic, and illness-related information, and sleep disturbances, MT and PA (see below). A neurologist assessed the degree of disability with the Expanded Disability Status Scale (EDSS; see below). The Review Board of the Esfahan University of Medical Sciences (Esfahan, Iran) approved this follow-up study, which was performed in accordance with the ethical principles laid down in the Declaration of Helsinki.

### Participants

Of the 23 patients assessed at disease onset (33), 18 agreed to participate in the follow-up study. The mean age was *M* = 34.29 years (SD = 3.21; 15 females and three male patients). The mean EDSS score was 2.05 (SD = 1.78). Participants and non-participants did not differ with regard to age, EDSS, gender, type of MS (relapsing remitting MS), treatment compliance, sleep disturbances, MT and PA scores at baseline (all statistical tests < 1.0, p's > 0.50). Current medications were as follows: Fingolimod: *n* = 5; Interferon beta-1a: *n* = 4; interferon beta-1b: *n* = 2; Natalizumab: *n* = 6; Mitoxantron: *n* = 1, without additional treatment. Medication remained unchanged throughout the 2 years. None of the patients had a relapse between disease onset and follow-up. All participants were diagnosed with MS, relapsing-remitting subtype (RRMS). Key eligibility criteria for the follow-up study included the participation in the baseline study, a diagnosis of multiple sclerosis (according to the 2010 revised McDonald criteria; (47); an EDSS (see below) score of 0–5.5 at screening (scores range from 0 to 10.0, with higher scores indicating a greater degree of disability); no documented clinical relapses within the previous 2 years or one clinical relapse within the year before screening; magnetic resonance imaging (MRI) of the brain showing abnormalities consistent with multiple sclerosis; and no neurologic worsening for at least 30 days before assessment.

### Measures

#### Expanded disability status scale

As in the baseline study, an expert and neurologist not otherwise involved in the study assessed the EDSS score (48). The EDSS is an accepted and widely used tool to objectively assess disability levels of patients with MS. The total score is ranked on a scale from 0 to 10, with increments of 0.5–1.0, and with higher scores reflecting higher levels of disability. EDSS steps 1.0–4.5 refer to people with MS who are fully ambulatory. EDSS steps 5.0–9.5 are defined by the impairment to ambulation. Meyer-Moock et al. (49) reported in their systematic review the high validity and reliability of the EDSS. They further concluded that the EDSS is suitable to describe the clinical status and the physical disability, and to monitor disease progression.

#### Mental toughness (MT)

As in the baseline study, MT was assessed with the Mental Toughness Questionnaire 48 (34). As described extensively elsewhere (33) the questionnaire consists of 48 items, which are aggregated to the following dimensions: challenge (e.g., “Challenges usually bring out the best in me”), commitment (e.g., “I don't usually give up under pressure”), emotional (e.g., “Even when under considerable pressure I usually remain calm”), and life control (e.g., “I generally feel in control”), interpersonal confidence (e.g., “I usually take charge of a situation when I feel it is appropriate”), and confidence in ability (e.g., “I am generally confident in my own abilities”). Evidence for the factorial validity of the MTQ48 has been reported in previous studies (35). Further, the MTQ48 has a high test–retest reliability and a high internal consistency. Items are anchored on 5-point Likert scales from 1 (strongly disagree) to 5 (strongly agree), with higher scores reflecting higher MT. Additionally, responses across items were summed to obtain an overall MT index (Cronbach's alpha = 0.80–0.87).

#### Physical activity (PA)

Again as extensively described in the previous study (33), physical activity was assessed with the short version of the International Physical Activity Questionnaire (IPAQ; for the description of the dimensions, see (50). The short (self-administered, seven-item) version of the IPAQ was used, asking about the time spent in PA over the last week. Minutes of sitting and walking, as well as moderate-intensity (walking not included) and vigorous-intensity activities were calculated for the past 7 days.

#### Sleep disturbances

As mentioned in the previous study (33), the Insomnia Severity Index (ISI; (51) was used to assess subjective sleep disturbances. This questionnaire consists of seven items to assess subjective insomnia. The items, answered on 5-point Likert-rating scales ranging from 0 (= not at all) to 4 (= very much), refer in part to the Diagnostic and Statistical Manual of Mental Disorders [DSM−5; American Psychiatric Association, (52)] criteria for insomnia by assessing difficulty in falling asleep, difficulties remaining asleep, early morning awakenings, impaired daytime performance, impaired daytime performance, low satisfaction with sleep, and worry about sleep. The higher the overall score, the more the participant is assumed to suffer from insomnia (Cronbach's alpha = 0.91). Valid and satisfactorily psychometric properties have been reported (e.g., (53). Further, (51) proposed the following categories: 0–7: no clinically significant insomnia; 8–14: subthreshold insomnia; 15–21: clinical insomnia (moderate severity); 22–28: clinical insomnia (severe).

### Statistical analysis

With a series of paired *t-*tests, we compared means of PA, MT, and sleep disturbances at disease onset and 2-year follow-up. Further, intra-class correlation coefficients were reported as an indicator of correspondence between the means. Next, with a series of Pearson's correlations, we examined the associations between the EDSS scores at follow-up and the mean differences of EDSS scores with PA, MT, and sleep disturbances. With a χ^2^-test, we explored if insomnia categories changed over time.

Across all analyses, the nominal level of significance was set at alpha ≤ 0.05. All statistical computations were performed with SPSS® 24.0 (IBM Corporation, Armonk NY, USA) for Apple Mac®.

## Results

### Scores of EDSS, sleep disturbances, physical activity, and mental toughness over time

Table 1 shows the descriptive statistics and an overview of all inferential statistical with regard to age, EDSS scores, PA, MT, and sleep disturbances, both at baseline (disease onset) and 2 years later.

Table 1Overview of descriptive and inferential statistical indices of socio-demographic variables, mental toughness, subjective sleep, and the EDSS score at baseline and 2 years later (*N* = 18).

**Table:** 

	**Baseline**	**2 years later**
Age (years)	32.09 (3.20)	34.29 (3.21)
EDSS score	0.81 (0.82)	2.05 (1.78)
Sleep disturbances	11.33 (3.73)	10.83 (3.71)
**MENTAL TOUGHNESS**		
Challenge	26.87 (3.56)	26.31 (3.09)
Commitment	38.89 (6.58)	35.63 (5.57)
**CONTROL**		
Control emotions	21.00 (4.26)	20.13 (3.90)
Control life	23.19 (5.08)	24.13 (4.47)
Control total	44.19 (7.96)	44.29 (6.96)
**CONFIDENCE**		
Confidence abilities	29.19 (6.01)	27.75 (5.41)
Confidence interpersonal	21.04 (3.46)	23.81 (4.27)
Confidence total	50.19 (6.59)	51.56 (4.78)
Overall score	159.94 (21.82)	157.75 (21.20)
**PHYSICAL ACTIVITY**		
Moderate physical activity (min/week)	550.56 (204.15)	515.00 (256.86)
Vigorous physical activity (min/week)	176.39 (109.97)	77.78 (45.66)
Walking (min/week)	371.94 (90.11)	1141.94 (355.04)
Sedentary behavior (min/week)	5529.72 (457.37)	4143.33 (874.74)

**Table:** 

	**Paired** ***t-*****tests**	**Cohen's d effect size**	**Intraclass coefficients**	**Confidence interval**
Age (years)	1.24	0.68 M	0.98	0.78–0.99
EDSS score	3.77^***^	0.89 L	0.81	0.51–0.93
Sleep disturbances	0.53	0.13 S	0.89	−0.09–0.85
**MENTAL TOUGHNESS**				
Challenge	0.82	0.17 S	0.80	0.42–0.93
Commitment	3.38^**^	0.53 M	0.90	0.72–0.97
**CONTROL**				
Control emotions	1.09	0.23 S	0.82	0.48–0.94
Control life	1.05	0.19 S	0.84	0.53–0.94
Control total	0.04	0.03 S	0.82	0.48–0.94
**CONFIDENCE**				
Confidence abilities	1.36	0.25 S	0.88	0.55–0.95
Confidence interpersonal	0.92	0.71 M	−0.16	−2.31–0.60
Confidence total	0.42	0.23 S	0.22	−1.24–0.73
Overall score	0.49	0.10 S	0.66	146.50–171.57
**PHYSICAL ACTIVITY**				
Moderate physical activity (min/week)	0.20	0.15 S	−0.02	−1.73–0.62
Vigorous physical activity (min/week)	1.15	1.17 L	−0.31	−2.50–0.51
Walking (min/week)	2.52^*^	2.98 L	−0.03	-1.76–0.61
Sedentary behavior (min/week)	1.85^+^	1.98 L	−0.12	−1.51–0.58

EDSS, Expanded Disability Status Scale;

+ p < 0.1;

* p < 0.05;

** p < 0.01;

*** *p < 0.001; degrees of freedom: always (17). S, small effect size; M, medium effect size; L, large effect size*.

EDSS scores significantly increased; in other words: there was a slight worsening in disability from virtually no disability (0.81) to a minimal impairment in the functional system (2.05).

**Figure 1:**
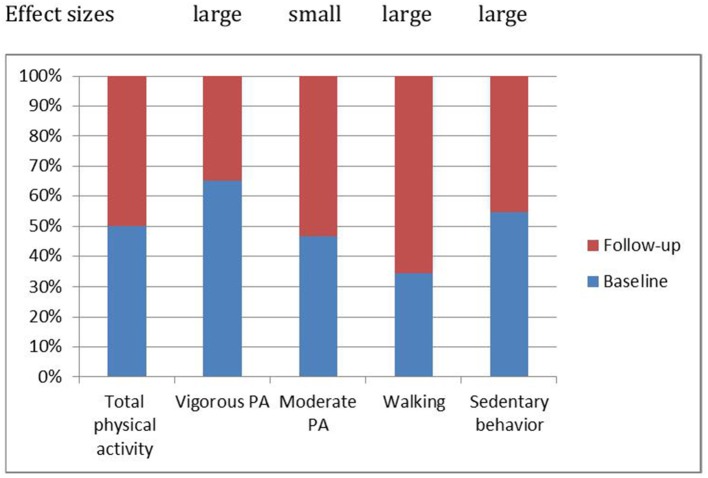
Patterns of physical activity changed over time: Compared to the baseline, 2 years later vigorous physical activity decreased, moderate physical activity remained stable, walking increased and sedentary behavior decreased.

With regard to PA, moderate PA remained unchanged, while vigorous PA decreased significantly (large effect size). On the flip side, walking increased significantly (large effect size), and sedentary time decreased also significantly over time (large effect size). Figure 1 shows the relative changes in overall PA and its components (moderate and vigorous PA; walking; sedentary time).

With regard to mental toughness, a significant decrease was observed in the Commitment dimension and a descriptive increase was observed in Confidence Interpersonal (with a medium effect size). All other dimensions of MT remained stable and unchanged (no statistical significant mean differences, high intra-class coefficients, and small effect sizes).

Sleep disturbances remained unchanged. This holds also true as insomnia categories did not change over time: Baseline: no insomnia: *n* = 5; subthreshold insomnia: *n* = 8; clinical insomnia (moderate severity): *n* = 5; Follow-up: no insomnia: *n* = 4; subthreshold insomnia: *n* = 5; clinical insomnia (moderate severity): *n* = 5; χ^2^(*N* = 18, df = 3) = 0.78, *p* = 0.89.

### Correlations between EDSS scores at follow-up, changes in EDSS scores, and PA, MT, and sleep

A series of Pearson's correlations were performed to calculate the associations between PA, MT, sleep, and EDSS scores at the 2-year follow-up, and the changes of EDSS scores over time. An increased EDSS score (that is, a higher impaired disability) was associated with a lower walking time (min/week): *r* = −0.63, *p* < 0.01).

## Discussion

The key findings of the present study are that 2 years after MS onset, vigorous PA decreased, moderate PA remained unchanged, walking increased, and sedentary time decreased. Further, both MT traits and sleep disturbances remained fairly stable and unchanged. In our opinion, the present pattern of the results expands upon previous research in that we showed that PA, MT, and sleep remained stable over 2 years after disease onset. Importantly, the total amount of PA did not decrease over time.

Four hypotheses and one research question were formulated and each of these is considered now in turn.

With the first hypothesis, we assumed that PA at disease onset was associated with PA 2 years later; data showed a rather sophisticated pattern: While vigorous PA decreased, moderate PA remained stable, walking increased and sedentary time decreased. Accordingly, the present pattern of results adds to the current literature in an import way: In MS, PA does not necessarily decrease, but changes in a specific pattern from vigorous to walking as a proxy of a more softer way of PA. Further and most interestingly, sedentary time decreased, also because such a decrease was against previous results (20). The quality of the data does not allow a deeper understanding as to why sedentary behavior decreased. Though highly speculative, it is conceivable that with the reduction of vigorous activity, the need to recover via sedentary behavior also decreased. Next, participants might have changed their general physical activity behavior due to the recommendations received at the study center: Specifically, they were encouraged to stay physically active, despite the diagnose of MS.

With the second hypothesis, we assumed that changes in PA would be related to disability, though data did not support this assumption: Changes in PA were not related to EDSS scores (at follow-up and changes of EDSS scores over time). Thus, our data are at odds with the results of Motl and McAuley (28) who observed a deterioration of PA as a function of increased disability. To explain these diverging results, we claim that patients in the present study showed rather small impairment (mean EDSS score: 2.05), or in other words: patients of the present study were only marginally impaired, and accordingly, from a statistical point of view, the low variance in EDSS scores most probably did not allow a higher variation in further dimensions such as PA, MT, or sleep.

With the third hypothesis, we expected that sedentary behavior would increase over time, but data confirmed the opposite: Two years later, sedentary time decreased significantly (large effect size), and accordingly, the present results are at odd with the assumption that sedentary behavior might increase in the first years after MS onset (cf. (20).

With the fourth hypothesis, we assumed that MT scores would decrease over time, though again data did not confirm this. Rather, MT scores remained fairly stable over time, or in other words: Challenge, Control (emotion, life, total), and Confidence (abilities, total) remained unchanged, while Commitment decreased, and Confidence (interpersonal) increased (medium effect size). Therefore, the pattern of results is more fine-grained.

The quality of the data does not allow a deeper understanding of such a specific pattern. In this view, there is no unanimous consent, if MT is a trait or a state: While some emphasize the stability of MT traits over time, which are understood as personality traits (34, 54, 55), for instance (56) claimed that MT is transient and state-dependent. Further, the concept of MT is interesting and promising, as it comprises a broad variety of psychological dimensions such as coping, self-esteem, emotion regulation, and social interactions (see (33) for a thorough review), all of which are crucial for coping with a chronic disease such as MS. For short, we claim that the present results expand upon previous literature in that we showed that personality states such as MT aspects seem to remain stable, at least within the first 2 years after disease onset.

With the research question we asked, if and to what extent sleep disturbances (i.e., sleep quality) changed over time; the answer was that subjectively assessed sleep disturbances remained unchanged, as neither means nor insomnia categories altered. Given the lack of comparable data, we claim that the stability of sleep disturbances (or sleep quality) over a time lapse of 2 year is astonishing, as patients were confronted with the diagnosis of a chronic disease. Accordingly, based on the hyperarousal model of sleep disturbances (57), one might have assumed a deterioration in subjective sleep. However, based on the present results, one might claim that patients' sleep seemed to be restoring. This assumption holds particularly true, as the mean score of sleep disturbances was about 11 points, which following the normative ranges of Bastien et al. (51) equal to a subthreshold insomnia.

Despite the encouraging results, the following limitations should be taken into account. First, the sample size was small, though, we focused on effect size calculations, which are not sensitive to sample sizes. Second, we assessed a rather specific sample of patients with MS; that is to say: The sample was prevalently female, young and at an early stage of disease. Accordingly, the sample does not reflect the broad variety of patients at older age, male patients, and patients at a higher degree of disability. Third, it is conceivable that unassessed latent factors might have biased two or more dimensions in the same or opposite direction; specifically, it is conceivable that the assessment of confounders such as depression, anxiety, daytime sleepiness, and fatigue may have conferred to different pattern of results. Fourth, the latter point holds particularly true for the burden of lesions. Fifth, sleep was only subjectively assessed, while objective sleep assessments would have allowed a deepened understanding of sleep continuity and sleep architecture characteristics. In this view, we acknowledge that further sleep issues such as hypersomnia, Restless Legs Syndrome (RLS), Periodic Limb Movements (PLMs), breathing-related sleep issues and medication-related sleep alterations were not taken into account. Accordingly, future studies should thoroughly assess typical sleep issues as mentioned above. Sixth, this holds also particularly true as regards the objective assessment of PA with activity trackers. In this view, seventh, in the present study, we did fully rely on patients' self-reports, while experts' ratings on dimensions of anxiety and depression would have strengthened the reliability of the pattern of results.

## Conclusions

Two years after disease onset, patients with MS reported a stable and favorable PA pattern, stable Mental toughness traits and unchanged and mild sleep disturbances. Such patterns of results should be encouraging for patients with MS, their relatives and the health care providers. Specifically, at the early stage of the disease, MS does not seem to be an obstacle for being physically active, nor did sleep or MT as a proxy of cognitive-emotional processes decrease in a dramatic way. Further longitudinal studies lasting more than 2 years are needed to estimate the long-term development of physical activity, sleep, and mental toughness as a proxy of psychological functioning.

## Author contributions

DSB, LE, VS, MG, JK, UL, EH-T, and SB: Designed the study; DSB, LE, and VS: Cared for data gathering and data entry; DSB, LE, MG, EH-T, and SB: Performed the statistical analyses; DSB, LE, MG, JK, UL, EH-T, and SB: Wrote the draft; SDB: Gathered the authors' comments; DSB, LE, MG, EH-T, and SB: Completed the final version of the manuscript. All authors agreed with the final version.

### Conflict of interest statement

The authors declare that the research was conducted in the absence of any commercial or financial relationships that could be construed as a potential conflict of interest.
